# Culturally Adapted Manual‐Assisted Psychological Intervention (CaMaPI) for Adolescents/Young People With a History of Self‐Harm and Suicidal Ideation in Nigeria: A Randomised Controlled Feasibility Trial

**DOI:** 10.1002/cpp.70098

**Published:** 2025-06-11

**Authors:** Dung Ezekiel Jidong, Tarela Juliet Ike, Maigari Yusufu Taru, Juliet Y. Pwajok, Charles Nnaemeka Nwoga, John Ezekiel Jidong, Shadrack B. Mwankon, Christopher Francis, Nusrat Husain

**Affiliations:** ^1^ Division of Psychology and Mental Health The University of Manchester Manchester UK; ^2^ Department of Sociology and Criminology Teesside University Middlesbrough UK; ^3^ Department of Psychiatry Jos University Teaching Hospital Jos Nigeria; ^4^ University of Jos Jos Nigeria; ^5^ The University of Greater Manchester Manchester UK; ^6^ Department of Psychology University of Jos Jos Nigeria

**Keywords:** adolescent, intervention, Nigeria, self‐harm, suicidal ideation, young people

## Abstract

**Background:**

Globally, suicide is the second leading cause of death for adolescents/young people aged 15–30‐years old. The mainstream care for these affected persons is mostly unsuccessful due to limited culturally appropriate care.

**Methods:**

This is a mixed‐methods randomised controlled feasibility trial design. A culturally adapted manual‐assisted psychological intervention (CaMaPI) was utilised to treat adolescents/young people with histories of self‐harm and suicidal ideation. CaMaPI is a manualised intervention consisting of 10 sessions, grounded in psychoeducation and cognitive behavioural therapy. Participants aged 18–29 were screened for self‐harm and suicidal ideation. *N* = 20 participants were randomised into CaMaPI (*n* = 10) or Treatment as Usual (TaU) (*n* = 10) groups. One focus group with *n* = 8 participants, and *n* = 3 individual interviews were conducted with the experimental group.

**Result:**

Satisfaction with intervention (CaMaPI, 100%; TaU, 50%). Reduction in self‐harm and suicidal ideation was higher in CaMaPI on the suicide and self‐harm scale at Md = 1.00 with z = −2.264, compared to TaU, Md = 3.00 with z = −0.378. Both groups showed no significant reduction in hopelessness. Emerging themes from the qualitative findings showed acceptance of self‐harm and suicidal ideation as a treatable condition, mood management and behaviour modification, alongside cultural appropriateness and positive experience of the CaMaPI.

**Conclusions:**

CaMaPI is feasible, culturally appropriate and acceptable in reducing self‐harm and suicidal ideation in adolescents/young people with histories of self‐harm and suicidal ideation in Nigeria. A fully powered randomised control trial is recommended to evaluate the clinical and cost‐effectiveness of CaMaPI compared with TAU.

**Trial Registration:** ClinicalTrials.gov (No. NCT06440031)

**Key Practitioner Message:**

- Suicide is one of the leading causes of death among 15‐ to 29‐year‐olds globally.
- Seventy‐three percent of all suicides and self‐harm happen in low‐ and middle‐income countries, including Nigeria.
- CaMaPI is acceptable, culturally appropriate and feasible for treating suicidal ideation and self‐harm behaviours in adolescents and young people.
- CaMaPI is manualised and delivered with minimal resources by trained clinical psychology researchers.

## 1 Introduction

Suicide and self‐harm are major public health concerns. Early adolescence constitutes the onset of suicidal ideation and self‐harm tendencies (Whitlock and Selekman 2014). For example, a study of the Canadian population showed that the average age of first occurrence of self‐harm and suicidal ideation is estimated at 13–15‐years old (Gardner et al. 2019; Nixon et al. 2008). The prevalence of self‐harm in adolescents is also between 12.1% and 18.8%, with females more likely to engage in self‐harm (Hawton et al. 2020). The World Health Organisation (WHO) (2024) data suggest that an estimated 20% of children and adolescents have a mental health condition, of which suicide is the second leading cause of death among those aged 15 to 30‐years old. The prevelence of self‐harm and suicidal ideation are more common in low‐ and middle‐income countries (LMICs) like Nigeria. Thus, 73% of global suicide and self‐harm happens in LMICs (WHO, 2024). Other studies have aimed to address the prevalence and correlation between self‐harm and suicidal ideation, and found that predisposing issues such as substance use (Animasahun and Animasahun 2016), self‐esteem (Uwaoma et al. 2023), including family and cultural factors (Omigbodun et al. 2008), play significant roles. For example, Omigbodun et al. (2008) conducted a study based on a survey of 1429 adolescents aged 10–17 years, and found that this cohort of young people from disrupted and polygamous families residing in urban areas had higher rates of suicidal ideation. For this study, ‘suicidal ideation’ is defined as persistent thoughts of dying by suicide due to psychological distress, while ‘self‐harm’ is defined as a deliberate injury to oneself (Harmer et al. 2022). We have also defined the age of adolescents and young people for the present study to be between 18–29 years. In Nigeria, it is apparent that factors such as religious and cultural bealiefs could potentially play an important role in adolescents and young people self‐harm and suicidal ideation. However, there is a dearth of literature surrounding cultural and context‐specific interventions for adolescents and young people with a history of suicidal ideation and self‐harm.

Despite the limited research attempts to provide culturally sensitive interventions for suicidal ideation and self‐harm for adolescents and young people, one study has used a culturally adapted manual‐assisted problem‐solving intervention for youth (YCMAP) with a history of self‐harm in Pakistan (Tofique et al. 2024), and thus, the YCMAP intervention was found to be essential and effective for adolescents, who are an important target population for the prevention of suicidal ideation, self‐harm and other mental health problems. In terms of culture‐specificity, Tofique et al.'s (2024) YCMAP may have different implications for Nigerian young people and their cultural context.

Culturally adapted manual‐assisted psychological intervention (CaMaPI), also known as YCMAP, is a psychological intervention based on the principles of cognitive behaviour therapy (CBT), including 10 sessions delivered over 3 months. The sessions are offered weekly, with each session lasting approximately 90 min. The intervention includes psychoeducation and a comprehensive cognitive behavioural assessment of the self‐harm attempt using virtual stories of four young people. CaMaPI is a cognitively oriented and problem‐focused therapy and is delivered as a group session to adolescents and young people with history of self‐harm and suicidal ideation (about eight‐ten persons per group) by trained clinical researchers (CRs) or therapists. The therapy focuses on current problems that contributed to the self‐harm episode. Assessment of coping strategies is carried out flexibly depending on the specific needs of the young persons. Training in assertiveness and anger management are offered to help young people develop resilience and cope with stress (Schmidt et al. 2015).

Studies on suicidal ideation within the Nigerian context tend to focus on understanding public attitudes and self‐harm prevention strategies. For example, a Jidong, Ike, Husain, et al. (2024) study comprising 562 nonclinical samples about their tolerance toward self‐harm and 18 interviewed participants, including patients with a history of self‐harm and suicidal ideation, caregivers and clinicians, found that substance use, perceived social isolation and rejection were considered predisposing factors for suicide and self‐harm. Subsequently, the few studies that have sought to test interventions among youth tend to focus on the role of media and self‐compassion in enhancing and preventing suicide among youth (Latha et al. 2020)—although this study was not based in Nigeria. However, a relevant study, which was based in Nigeria, only focused on enhancing mental health or the use of counselling strategies to address suicidal ideation and depression among students in tertiary institutions (Fadipe and Okesina 2024). Thus, the gap remains as self‐harm and suicidal ideation can affect any young adolescent regardless of whether they are students, thereby limiting its reach to broader adolescents/young people groups.

Whilst the preceding studies provide insight into the prevalence and correlates to engaging in suicidal behaviours, alongside few interventions to address the problem, there is a significant gap in testing the psychosocial interventions among adolescents and young people in countries like Nigeria.

Our study attempts to fill this gap by specifically making an original and significant contribution by testing the feasibility of the CaMaPI for treating adolescents and young people with a history of self‐harm and suicidal ideation in Nigeria. More specifically, the research question we seek to answer is:

- How feasible, acceptable and culturally appropriate is the CaMaPI for adolescents/young people with a history of self‐harm and suicidal ideation compared to Treatment As Usual (TAU) in Nigeria?

To reiterate, within the context of this study, the ages of ‘adolescents and young people’ are operationally defined as individuals between 18‐ and 29‐years old. This is because suicide is one of the leading causes of death among 15‐ to 29‐year‐olds globally, and 73% of all suicides happen in LMICs, including Nigeria (WHO 2024).

## 2 Methods

### 2.1 Design

The present study is informed by a randomised controlled trial feasibility design, comparing the CaMaPI + TAU with TAU alone. RCT is often considered the gold standard for testing psychological interventions due to its rigour and replicable methodological approach (Skivington et al. 2021).

### 2.2 Ethics

The study received ethical approval (no. JUTH/DCS/IREC/127/XXXI/2702) from the Jos University Teaching Hospital (JUTH) Research Ethics Committee, Nigeria.

### 2.3 Recruitment

Adolescents/young people with a history of self‐harm and suicidal ideation were recruited from the Suicide Prevention and Response Unit of the JUTH. The study flyers were distributed to potential recruitment places such as mosques, churches and community venues/organisations.

The inclusion criteria for participants to take part in the study comprised adolescents/young people with a history of self‐harm and suicidal ideation between the ages of 16 and 30 who presented to the participating services and emergency departments or were admitted after an episode of self‐harm or suicidal ideation to the Suicide Prevention and Response Unit of JUTH or self‐referrals.

Exclusion criteria include persons with severe mental illness (such as psychotic disorder), conditions limiting engagement with assessment/intervention, temporary residents unlikely to be available for follow‐up and patients with chronic comorbidities unable to provide informed consent.

### 2.4 Participants Demography

In total, 20 participants were recruited for the study. Of the *N* = 20 total participants recruited for the study, *n* = 13 were females, while the other *n* = 7 were males. For the level of education, six indicated having attained a secondary level of education, whilst the other 14 indicated tertiary‐level education. In terms of employment, *n* = 8 were employed at the time of the intervention, whilst the other *n* = 12 were unemployed. For religious demography, *n* = 4 were Muslim, while *n* = 16 were Christians. The participants' age range was between 18 and 29 years.

### 2.5 Randomisation

Randomisation was conducted using Microsoft Excel. Participants who met the study's inclusion criteria were randomised into the CaMaPI + TAU or the TAU alone group. Based on the list of eligible participants, a value of 0 or 1 was randomly generated using the RANDBETWEEN function in Excel, thereby choosing 0 and 1 as the range. All participants with 0 were assigned to the controlled group (TAU), and all participants with 1 were assigned to the experimental group (CaMaPI). Adopting a single sequence of random assignments in which participants were assigned based on a 1:1 ratio allowed for less bias and equal chances of allocating all participants to either experimental or controlled groups (Kim and Shin 2014). Thus, all participants had equal chances of receiving either the CaMaPI + TAU or the TAU alone. All participants were blinded to whether they were assigned to experimental (CaMaPI) or controlled (TAU) groups. Similarly, the statistician analysing results was equally blinded. However, CRs delivering the CaMaPI intervention were not blinded due to their involvement in the cultural adaptation, study protocol development and the delivery simulation exercises.

### 2.6 Assessments

Participants were assessed using the Scale for Suicidal Ideation (Beck et al. 1979), Hopelessness Scale (Marshall et al. 1992) and Suicide and Self Harm Evaluation Scale (Tyson et al. 2016). Participants were assessed at the baseline, end of intervention and 3‐months postintervention. The study's primary outcome measures were to examine the cultural appropriateness, acceptability and feasibility of the CaMaPI intervention, which was assessed using the Service Satisfaction Scale (Ike et al. 2023). In terms of the secondary outcome measures, with assessments at baseline, end of intervention at and 3‐months post‐intervention, the scales used for assessment include the Scale for Suicidal Ideation, Hopeless Scale and Suicide and Self Harm Evaluation Scale.

### 2.7 Cultural Adaptation of CaMaPI

We adopted an ‘Iterative Model of Co‐adaptation’ (IMC) (Jidong et al. 2023) for the CaMaPI manual to ensure it is culturally appropriate and suitable for adolescents and young people in Nigeria. The IMC process includes thoroughly reviewing the CaMaPI manual (see Table 1) in community involvement and engagement (CEI) events. The CaMaPI manual adaptation was well‐crafted to understand the cultural perspectives of self‐harm and suicidal ideation, including factors such as idioms of mental distress and their perceived causes leading to self‐harm and suicide. The adaptation process also explored context‐specific factors such as cultural and religious beliefs, including linguistic constructions of realities such as cultural expectations of spoken and nonspoken languages related to mental distress.

**TABLE 1:** CaMaPI sessions and content.

Sessions	CaMaPI content
1	Getting started psycho education: What is self‐harm?
2	What to do in a crisis
3	Cognitive behaviour therapy emotions and feelings
4	Motivation to change
5	Negative automatic thoughts (NATs)
6	Core beliefs
7	Coping strategies
8	Assertiveness continuing journey
9	Anger management
10	Family session and Intervention completion ceremony

The CaMaPI adaptation was also informed by our mixed‐methods study (Jidong, Ike, Husain, et al. 2024) with *n* = 18 interviewed key stakeholders comprising (*n* = 11 clinicians, *n* = 5 patients with a history of self‐harm and suicide ideation and *n* = 2 caregivers) and *n* = 562 non‐clinical sample was surveyed about their tolerance toward self‐harm. We also conducted a systematic review of interventions for suicidal ideation and self‐harm in Africa to understand the interventions, strengths and weaknesses prior to engaging in the present study (Jidong, Ike, Murshed, et al. 2024). All learning and feedback from foundational works (i.e., mixed‐methods study, systematic review, community involvement and engagement, presentations in seminars, conferences and workshops) informs the CaMaPI manual's adaptation, clinical value, practical utility and how the intervention could potentially be integrated into local care systems following fully powered randomised controlled trails.

### 2.8 Interventions

**FIGURE 1:**
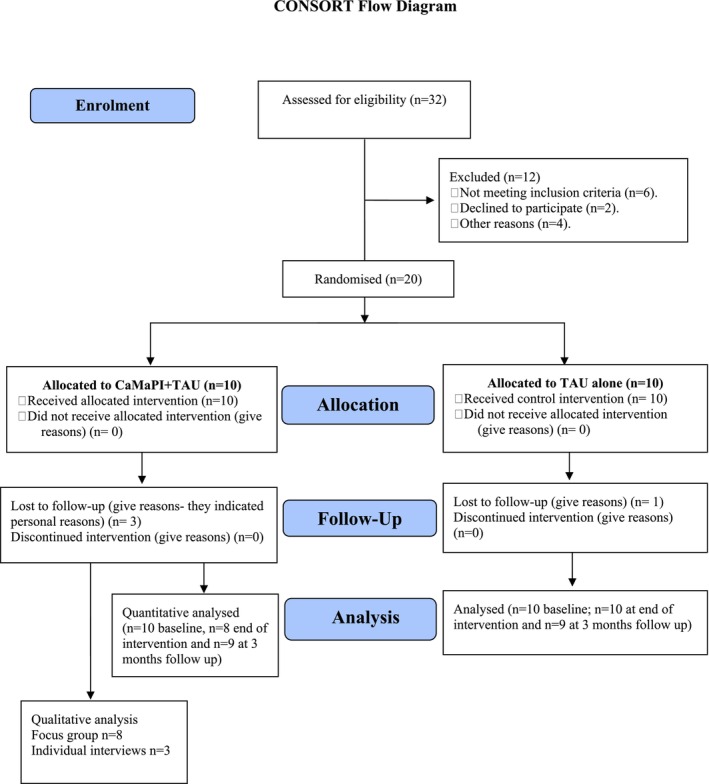
CONSORT flow diagram. Figure 1 shows a flow diagram of participants' recruitment, randomisation, retention and analysis.

*N* = 20 eligible participants were recruited (see Figure 1) and randomly assigned to one of two groups: Group 1, Experimental (*n* = 10), received CaMaPI + TAU, which consisted of a manually assisted brief psychological intervention based on the principles of cognitive behaviour therapy (CBT). The intervention includes psychoeducation and a comprehensive cognitive behavioural assessment of the suicidal ideation and self‐harm attempt using virtual stories of four young people and was delivered in 10 sessions (90 min each) on weekly basis (see details of CaMaPI sessions and content in Table 1). For the experimental group, the 10 sessions are the standard recommended duration for brief psychological interventions (Branquinho et al. 2021). Due to CaMaPI being manualised, sessions were delivered by trained clinical psychologists, supervised by the Principal Investigator (DEJ), senior clinical psychologist (JYP) and psychiatrist (MYT), every week.

The intervention was delivered in the Suicide Prevention and Response Unit of the JUTH. To avoid contamination, dedicated CRs facilitated the delivery of CaMaPI programme.

Group 2: Controlled (*n* = 10) received TAU alone—this is the participating service provider's regular treatment routine for the adolescents and young people diagnosed or presented with cases of suicidal ideation and self‐harm. The TAU is the standard patient care pathway that includes routine assessment, management and antidepressant prescriptions.

It is pertinent to note that two participants were lost to follow‐up in the experimental group, and personal reasons were cited as the basis for not completing the survey. In the control group, only one participant was lost to follow‐up, citing personal reasons.

### 2.9 Clinical Research Assistant Training

The study's intervention was delivered by clinical researchers (CRs) with MSc level certification in Clinical Psychology, who received (a) 1 day of intensive trainers' training on how to deliver the CaMaPI and also received weekly 1 h of CaMaPI simulation exercise with imaginery participants for each session before delivering the actual intervention. This weekly supervision was designed to ensure the intervention was delivered as per the study's protocol, maximum fidelity and the intervention's with alignment with global best practices.

### 2.10 Data Analyses

#### 2.10.1 Quantitative Analysis

*N* = 20 participants were recruited for the study, and data were analysed to determine the study's primary outcome measures, which is the CaMaPI's feasibility, cultural appropriateness and acceptability. This was measured using participants' satisfaction with the intervention scales (Ike et al. 2023). For secondary outcome measures, an inferential statistic using the Wilcoxon sign rank test of analysis was conducted to determine the intervention's preliminary clinical effectiveness in treating suicidal ideation and self‐harm among adolescents and young people. This non‐parametric statistical model was most appropriate due to data skewness across experimental and controlled groups (Sedgwick 2015).

#### 2.10.2 Qualitative Analysis

One focus group comprising *n* = 8 participants and *n* = 3 individual interviews was conducted with the CaMaPI experimental group participants. The interviews were in‐person, recorded using Microsoft Teams and an audio recording device. Qualitative data collection stopped upon saturation, when no new findings were being identified. All interviews were recorded, transcribed verbatim and analysed using an interpretative phenomenological analysis (IPA). This analytical lens represents the most appropriate qualitative method for examining participants' lived experiences of self‐harm, including suicidal ideation and engagement with the interventions. All participants' identifiable information, such as their names in individual interviews and focus group discussions, was anonymised to retain the IPA ideographic nuances in the dataset. The findings section integrates relevant data verbatim from the transcripts to support emerging themes. IPA theoretical features, including phenomenology, single‐ and double‐hermeneutics and idiographics (Shinebourne 2011; Smith 2017), which underpinned the data analysis, analytical commentaries and the interpretations of findings. In this context, the phenomenon of interest is the participants' experiences of the CaMaPI for their histories of self‐harm and suicidal ideation. The hermeneutics involved the researchers' experiential roles in examining the participants' efforts within the dataset as they made sense of their ideographic experiences that are peculiarly related to the CaMaPI for self‐harm and suicidal ideation. The study's qualitative arm helped capture additional rich datasets associated with participants' experiences of the intervention from their lived perspectives in ways that could not have been possible using structured psychometric tools or questionnaires (Jidong, Ike, Husain, et al. 2024). To ensure anonymity, we deleted all participants' names, including any identifiable information.

## 3 Results

### 3.1 Results: Primary Outcome Measures on Satisfaction With the Intervention

As shown in Table 2 above, overall, the experimental group demonstrated a higher level of satisfaction with the acceptability of the intervention at 100% when compared to the control group at 50%. The experimental group also showed higher satisfaction with the perceived effectiveness of the intervention, which was 100%, compared to the control group, which was 80% not sure, 10% indicating its perceived effectiveness. A plausible explanation as to why this may be the case could be attributed to the intervention's content on cognitive behavioural therapy and psychoeducation, which helps reduce negative thinking, low self‐esteem and addresses self‐harm tendencies and suicidal ideation. The benefit of the intervention was also reported strongly in the experimental group at 62%, suggesting they will recommend the intervention to others when compared to just 10% in the control group, indicating similar intentions. At the end of the intervention, while only *n* = 8 participants in the experimental group completed the survey, with two citing personal reasons for not completing the survey, we still recorded greater satisfaction with the intervention compared to the control group, where all *n* = 10 completed the survey. Overall, the result indicated that the CaMaPI is acceptable, feasible and culturally appropriate for treating self‐harm and suicidal ideation with a higher satisfaction rate across all indices when compared to the treatment as usual control group.

**TABLE 2:** Showing the differences between the acceptability scores of the Service Satisfaction Scale for CaMaPI + TAU and TaU alone group at 12 weeks end of intervention survey.

S/N	Survey questions	CaMaPi (*Exp*) *n* = 8					TaU (*Control*) *n* = 10				
		0	1	2	3	4	0	1	2	3	4
1.	Please rate your satisfaction on the acceptability of the intervention. 0 = unacceptable; 1 = slightly unacceptable; 2 = not sure; 3 = slightly acceptable; 4 = acceptable.					100	10		40		50
2.	Please rate your satisfaction with the effectiveness of the intervention. 0 = not at all effective; 1 = ineffective; 2 = not sure; 3 = effective; 4 = very effective.					100		10	80	10	
3.	How would you rate the quality of the intervention? 0 = worst approach; 1 = less quality; 2 = not sure; 3 = high quality; 4 = best approach.				75	25			70	20	10
4.	How would you rate your satisfaction with the intervention? 0 = not satisfied at all; 1 = not satisfied; 2 = not sure; 3 = satisfied; 4 = very satisfied.				12	88			10	70	20
5.	Would you recommend the intervention to others? 0 = definitely no; 1 = no; 2 = not sure; 3 = yes; 4 = definitely yes.				38	62		10	60	20	10

The Wilcoxon sign rank test analysis for the experimental group (CaMaPI+TAU), as shown in Table 3, highlights a difference in the level of reduction in suicidal ideation at baseline when compared to the end of the intervention and three follow‐ups. This was denoted in the decrease in suicidal ideation from baseline (*Md* = 3.00) to end of intervention (*Md* = 1.00) with *p =* 0.014 and at 3‐month follow‐up (*Md* = 1.00) with *p =* 0.024. For the Hopelessness scale, the finding denotes no statistically significant reduction in hopelessness from *Md* = 2.00 at baseline to *Md* = 3.00 with *p =* 0.52 at the end of the intervention and *Md* = 3.00 with *p =* 0.46 at 3‐month follow‐up. In essence, while it appears the CaMaPI was promising in reducing suicidal ideation, it appears not to have impacted their level of hopelessness. Concerning the suicide and self‐harm evaluation, we found no difference in the intervention on the risk of self‐harm from baseline to *Md* = 1.00 to the end of intervention *Md* = 2.00 with similar scores at 3‐month follow‐up.

**TABLE 3:** Descriptive table showing medians and categories using Wilcoxon sign rank test scores across timepoints for CaMaPI + TAU (experimental group) at baseline, end of and 3‐month postintervention.

	Baseline	EOI	*z*	*p*	Baseline	3MFU	*z*	*p*
	*Md*	*Md*			*Md*	*Md*		
			Baseline * EOI				Baseline * 3MFU	
**SSI**	3.00	1.00	−2.456	0.014	3.00	1.00	−2.264	0.024
**HS**	2.00	3.00	−1.947	0.052	2.00	3.00	−1.994	0.046
**SASHES**	1.00	2.00	−2.428	0.015	1.00	2.00	−2.460	0.014

Abbreviations: HS, Hopelessness Scale; SASHES, Suicide and Self Harm Evaluation Scale; SSI, Scale for Suicidal Ideation.

In the controlled group (see Table 4), we observed no reduction in the level of suicidal ideation and self‐harm tendencies based on data from the scale for suicidal ideation from *Md* = 3.00 at baseline to *Md* = 3.00 at the end of intervention with *p =* 0.096 and at 3 months, *Md* = 3.00. Compared to the experimental group, the latter performed better, with *Md* = 1.00 at baseline and *Md* = 1.00 at the end of the intervention. In terms of hopelessness, we found no changes in the control group; instead, there was a slight increase in hopelessness from the baseline data at *Md* = 2.00 to the end of the intervention at *Md* = 2.50. At 3 months of follow‐up data, the level of hopelessness for the control group seems to have reverted to the baseline state at *Md* = 2.00. Finally, in terms of the suicide and self‐harm evaluation scale, similar findings show no changes from baseline *Md* = 2.00, and at the end of the intervention, *Md* = 2.00. At 3‐month follow‐up, the risk of self‐harm for the control group increased to *Md* = 3.00. The qualitative findings below provide insight into why we found more promising and positive outcomes with the CaMaPI group compared to the TAU.

**TABLE 4:** Descriptive table showing medians and categories using Wilcoxon sign rank test scores across timepoints for TAU alone (controlled group) at baseline, end of and 3‐month postintervention.

	Baseline	EOI	*z*	*p*	Baseline	3MFU	*z*	*p*
	*Md*	*Md*	Baseline * EOI		*Md*	*Md*	Baseline * 3MFU	
**SSI**	3.00	3.00	−1.667	0.096	3.00	3.00	−0.378	0.705
**HS**	2.00	2.50	−2.71	0.023	2.00	2.00	−1.342	0.180
**SASHES**	2.00	2.00	−1.414	0.157	2.00	3.00	0.000	1.000

Abbreviations: HS, Hopelessness Scale; SASHES, Suicide and Self Harm Evaluation Scale; SSI, Scale for Suicidal Ideation.

### 3.2 Qualitative Findings

The qualitative findings were based on participants' histories of self‐harm, suicidal ideation and their experience of the CaMaPI. Based on analyses underpinned by IPA, the following main themes were reported, including (a) acceptance of suicide and self‐harm as a treatable condition, (b) mood management and behaviour modification and (c) cultural appropriateness and positive experience of the intervention delivery. The IPA theoretical elements underpinned the analytical commentaries, which followed each extract of qualitative data verbatim in the results section.

#### 3.2.1 Acceptance of Suicide and Self‐Harm as a Treatable Condition

Self‐harm and suicidal ideation come with significant psychological distress, which affected persons often struggle to comprehend, by implication inhibiting their help‐seeking behaviour. A common pattern in the participant's lived experiences, as denoted in the dataset, was the perceived role of initially not accepting suicidal ideation as a condition that is real or capable of being treated. However, following engagement with the intervention, we saw an initial shift in perspective. Talking about this issue, one female participant said:

My experience is that this intervention is helping and gives us more experience and understanding of coping with the challenges that this disease that happened to us is real. It's made me understand that it's real. I used to think before it's just something like a magician […] It made me understand that it is real and that you can treat it. (Abokiya, a 20‐year‐old female)

On a similar note, another male participant whilst commenting on the intervention said:

My view is that when, [ … ] I am looking like saying that I like to kill myself, [ … ] some people will talked say you no dey well [unwell]? I will say I am well. When they bring this brief information and teaching[CaMaPI], it shows us something. […] before I wanted to kill myself because of this clinical depression, [but now referring to the CaMaPI] it is helpful, I see I am getting well and I understand‐ I understand that you must know how to converse with your parent and other relatives. Before I could not speak, I just stood like a stone […] when I started this [CaMaPI] and taking the medication, I started a conversation. (Oremii, a 24‐year‐old male)

The above participants' extract highlighted a significant pattern in which, when making sense of their experience of suicidal ideation, it was initially perceived it as an unreal condition. Phrases such as ‘I think before it's just something like a magician’ underscore a concerning trend that indicates a poor understanding of social ideation and self‐harm tendencies. The implication is that it risks downplaying the adverse effects of this condition, potentially worsening its outcome. However, following engagement with the intervention, the participants understood it and came to terms with it as a condition that could be managed through available treatment options such as CaMaPI.

#### 3.2.2 Mood Management and Behaviour Modification

One of the unintended consequences of suicidal ideation and self‐harm is its impact on the mood of those affected. Such mood could manifest through anger outbursts, which further exacerbate the condition. In recounting their lived experience of the intervention, the participant commented on its usefulness in enabling them to manage such outbursts through understanding positive behaviour modification techniques and how to engage in conversation with others. Onemale participant in recounting his experience said:

It is useful for me to understand conversations with different people. I like how you said how to interact with people, sometimes it will be like speaking to people with anger. Whenever people speak to me, I am just angry. They are speaking to me, and I will just be shouting […], but now [after CaMaPI] it is not so. (Wotofong, a 19‐year‐old male)

Another male participant, whilst recounting his experience, said:

The case study is very instructive; you can pick from the case study what your challenge is and learn from them. […] It is nice knowing that we come, and we are given the opportunity to speak, and it shows that we are taking away our inferiority complex. I think I enjoy the anger management. The anger management was really helpful because this have been my major problem and now, I think I am not suffering from an anger problem anymore. (Worok, 21‐year‐old female)

Conversations are integral to day‐to‐day interaction with others. The tone of conversation relies on emotional processes such as happiness or anger. Anger remains a significant challenge in coming to terms with suicidal ideation and self‐harm tendencies. Such feelings of anger could be further informed by a number of factors, including low self‐worth, being unable to process the social or self‐harm tendencies or even managing the feeling. As the preceding extract shows, the participant engaging with the intervention felt an increased sense of self‐worth similar to emotional intelligence in their day‐to‐day conversations with significant others.

#### 3.2.3 Cultural Appropriateness and Positive Experience of the Intervention Delivery

A recurrent pattern in the participant's lived experiences was the perceived cultural appropriateness and satisfaction with the CaMaPI delivery. This was construed from diverse perspectives, including how the intervention helped harness some of the rich cultural values often taken for granted, such as associating with the right people and inner peace management techniques. Commenting on this, one male participant said:

I like how you gave us something in the training[CaMaPI], and we wrote, and we still went home to do an assignment and understand something, that helped us know more helpful things. […] it is associated with my culture […] assuming like one, how to make your heart to be in peace, two, to make yourself stronger and good to leave bad people and to leave bad habits and to come out in good piece. (Yop, 18‐year‐old female)

In the preceding extract, the Yop denotes positive experiences engaging with the intervention and how she felt it resonates with her cultural values in ways that instil positive norms. Such positive norms and values were perceived to be culturally informed, were also seen to shape her experiences of the intervention and its usefulness in helping her address her unhelpful company and toxic associates, exacerbating self‐harm and suicidal ideation tendencies. Another female participant, whilst commenting on the CaMaPI training content, also said:

The training materials are well selected. They are well selected in the sense that the topics are very captivating, and it gives us the room to see what we have not seen before and to learn a lot. So, the materials are very ok. (Nvou, a 20‐year‐old female)

Again, the content of the intervention was perceived in a positive light. In making sense of Nvou's experience recounts the level of enlightenment received, which, for her, represents the unveiling of helpful information that she was previously unaware of as it relates to her condition. Such information was received with a high fascination, as indicated in her words, ‘very captivating’. Another participant, Gyang (an 18‐year‐old male), summed up his own experience of the rich topic delivered, said: ‘The training material is effective, and it is working. I can see my whole life changing’. The extract denotes how the information shared was perceived as beneficial in informing the perceived efficacy of the intervention in having positive benefits, especially as it relates to the primary aim of addressing self‐harm and social ideation.

## 4 Discussion and Conclusion

The primary aim of this study was to examine the feasibility, cultural appropriateness and acceptability of CaMaPI programme for treating adolescents and young people with a history of self‐harm and suicidal ideation at the Suicide Prevention and Response Unit of the Jos JUTH, Nigeria. Based on the primary outcomes, quantitative findings denoted higher satisfaction in the CaMaPI + TAU (experimental group) compared to the TAU (controlled group). The CaMaPI group also reported a good level of engagement and perceived effectiveness of the intervention compared to the controlled group. The preceding findings imply that the CaMaPI is feasible, culturally appropriate and acceptable for treating adolescents/young people with histories of self‐harm and suicidal ideation when compared to the TAU in Nigeria. The reason for the differences could be attributed to the robust design of the CaMaPI programme of activities, including the cultural adaptation of the intervention to ensure it helps embed the cultural values and context‐specific needs of the participants. It is also partly attributed to the engagement and treatment outcomes that improved their mental health.

The cultural appropriateness of CaMaPI in treating self‐harm and suicidal ideation resonates with a previous similar study. For example, Tofique et al.'s (2024) study comprised *n* = 684 participants, aged 12–18 years, presenting to general clinicians/physicians' emergency room after self‐harm or self‐referrals. Compared with TAU, the study tested the effectiveness of 8–10 individual intervention sessions delivered over 3 months. The study found reduced repetition rates of self‐harm, suicidal ideation, hopelessness and psychological distress in the intervention group arm as compared with TAU. It is crucial to note that these studies were conducted with South Asian populations; however, they showed promising results on the potential of CaMaPI in addressing self‐harm and suicidal ideation among the identified population.

Furthermore, findings from the study's secondary outcome measures showed higher reductions in self‐harm tendencies and suicidal ideation based on data from the suicide and self‐harm evaluation scale for the CaMaPI + TAU group compared to the TAU alone group. However, we found no improvement across both groups regarding the level of hopelessness. Despite this, the CaMaPI intervention was promising as it relates to self‐harm and suicidal ideation. Insights from the qualitative findings further found that the CaMaPI intervention provided the participants with rich knowledge, which aided their acceptance of suicide and self‐harm as treatable conditions, mood management and behaviour modification, cultural appropriateness and positive experience of the intervention delivery. All of these were informed by the integration of healthy thinking patterns underpinned by the theory of cognitivism (Sidik 2020). Thus, highlighting the positive benefit of their engagement with the CaMaPI programme.

### 4.1 Limitations

The main limitation of the present study is the small sample size, which was not statistically powered to detect the CaMaPI's clinical efficacy and effectiveness of treating self‐harm and suicidal ideation among adolescents and young people in Nigeria. The small sample size is due to the feasibility of the study design and limited resources at the time the study was conducted. As such, it is recommended that future studies conduct fully powered trials comparing CaMaPI with routine TAU. Another major limitation is the specific focus on one state in Nigeria, given the country's multi‐ethnic and cultural diversity. Future studies should conduct fully powered trials across Nigeria's six geopolitical zones while considering cultural diversity.

### 4.2 Study's Original Contribution and Clinical Implication

Self‐harm and suicidal ideation are significant global health challenges that impact mostly adolescents and young people. This study's intervention has made an original contribution through testing the CaMaPI treatment programme for adolescents and young people in Nigeria. By extension, this contributes to mitigating the disease burden of self‐harm/suicidal ideation and reducing such tendencies in the cohort of the beneficiaries in Nigeria. The CaMaPI  also contributed toward the United Nations Sustainable Development Goals (SDGs), such as SDG‐3—which is geared at good health and well‐being for all especially in LMICs like Nigeria with limited mental health workforce and poor access to culturally relevant care; SDG‐10—focusing on addressing inequality and inequity in the current state of mental health provision for adolescents and young people affected by self‐harm and suicidal ideation; and SDG‐17 on establishing global partnership and collaboration.

## 5 Conclusion

Self‐harm and suicidal ideation constitute major public health concerns that significantly affect adolescents and young people. Studies on the prevalence and correlation between self‐harm and suicidal ideation show that issues such as substance use, self‐esteem, including family and cultural factors all play significant roles in exacerbating self‐harm tendencies and suicidal ideation. In Nigeria, access to culturally appropriate care is scarce and further impacted by poor socio‐economic conditions, stigma, poor awareness of mental health services and religious beliefs. However, CaMaPI programme is promising and has shown to be feasible, culturally appropriate, acceptable and helpful in reducing self‐harm tendencies and suicidal ideation. The qualitative dataset of the adolescents and young people's experiences of CaMaPI also showed positive behaviour management, modification of negative thought processes and positive experiences with the intervention. In essence, a fully powered RCT is recommended to evaluate the clinical and cost‐effectiveness of CaMaPI compared with the TAU.

## Ethics Statement

The Jos University Teaching Hospital Research Ethics Committee approved the study. It was conducted in accordance with the institutional requirements and the Declaration of Helsinki. All participants provided their written informed consent prior to participating in this study.

## Conflicts of Interest

N.H. is a past Trustee of the Pakistan Institute of Living and Learning (PILL), Abaseen Foundation UK, Lancashire Mind UK, and Manchester Global Foundation (MGF). He is an executive member of the Academic Faculty at the Royal College of Psychiatrists, London, and an NIHR Senior Investigator. He has attended educational events organised by various pharmaceutical industries. N.H. and D.E.J. are also affiliated to the Global Centre for Mental Health Inequalities, Mersey Care NHS Foundation Trust, Liverpool, UK. D.E.J., S.B.M. and J.E.J. are trustees of the Dung Jidong Foundation (DJF). All other authors declare no conflicts of interest.

## Data Availability

Research data are not shared.

## Appendix 1 CONSORT 2010 Checklist of Information to Include When Reporting a Randomised Trial^a^

**Table:**

Section/topic	Item no.	Checklist item	Reported on page no.
**Title and abstract**	1a	Identification as a randomised trial in the title	
	1b	Structured summary of trial design, methods, results and conclusions (for specific guidance, see CONSORT for abstracts)	
**Introduction**			
Background and objectives	2a	Scientific background and explanation of rationale	
	2b	Specific objectives or hypotheses	
**Methods**			
Trial design	3a	Description of trial design (such as parallel and factorial) including allocation ratio	—
	3b	Important changes to methods after trial commencement (such as eligibility criteria), with reasons	
Participants	4a	Eligibility criteria for participants	
	4b	Settings and locations where the data were collected	
Interventions	5	The interventions for each group with sufficient details to allow replication, including how and when they were actually administered	
Outcomes	6a	Completely defined prespecified primary and secondary outcome measures, including how and when they were assessed	
	6b	Any changes to trial outcomes after the trial commenced, with reasons	—
Sample size	7a	How sample size was determined	
	7b	When applicable, explanation of any interim analyses and stopping guidelines	—
Randomisation:			
Sequence generation	8a	Method used to generate the random allocation sequence	
	8b	Type of randomisation; details of any restriction (such as blocking and block size)	—
Allocation concealment mechanism	9	Mechanism used to implement the random allocation sequence (such as sequentially numbered containers), describing any steps taken to conceal the sequence until interventions were assigned	
Implementation	10	Who generated the random allocation sequence, who enrolled participants and who assigned participants to interventions	
Blinding	11a	If done, who was blinded after assignment to interventions (for example, participants, care providers, those assessing outcomes) and how	
	11b	If relevant, description of the similarity of interventions	
Statistical methods	12a	Statistical methods used to compare groups for primary and secondary outcomes	
	12b	Methods for additional analyses, such as subgroup analyses and adjusted analyses	
**Results**			
Participant flow (a diagram is strongly recommended)	13a	For each group, the numbers of participants who were randomly assigned, received intended treatment and were analysed for the primary outcome	
	13b	For each group, losses and exclusions after randomisation, together with reasons	
Recruitment	14a	Dates defining the periods of recruitment and follow‐up	
	14b	Why the trial ended or was stopped	
Baseline data	15	A table showing baseline demographic and clinical characteristics for each group	
Numbers analysed	16	For each group, number of participants (denominator) included in each analysis and whether the analysis was	
		by original assigned groups	
Outcomes and estimation	17a	For each primary and secondary outcome, results for each group, and the estimated effect size and its precision (such as 95% confidence interval)	
	17b	For binary outcomes, presentation of both absolute and relative effect sizes is recommended	—
Ancillary analyses	18	Results of any other analyses performed, including subgroup analyses and adjusted analyses, distinguishing prespecified from exploratory	
Harms	19	All important harms or unintended effects in each group (for specific guidance, see CONSORT for harms)	
**Discussion**			
Limitations	20	Trial limitations, addressing sources of potential bias, imprecision and, if relevant, multiplicity of analyses	
Generalisability	21	Generalisability (external validity and applicability) of the trial findings	
Interpretation	22	Interpretation consistent with results, balancing benefits and harms, and considering other relevant evidence	
**Other information**			
Registration	23	Registration number and name of trial registry	
Protocol	24	Where the full trial protocol can be accessed, if available	
Funding	25	Sources of funding and other support (such as supply of drugs), role of funders	

^a^ We strongly recommend reading this statement in conjunction with the CONSORT 2010 Explanation and Elaboration for important clarifications on all the items. If relevant, we also recommend reading CONSORT extensions for cluster randomised trials, noninferiority and equivalence trials, nonpharmacological treatments, herbal interventions and pragmatic trials. Additional extensions are forthcoming: for those and for up to date references relevant to this checklist, see www.consort‐statement.org.

^b^ Please note that some items have been marked as not applicable because it does not apply to the present study.
